# Landscape Characteristics Affecting Spatial Patterns of Water Quality Variation in a Highly Disturbed Region

**DOI:** 10.3390/ijerph16122149

**Published:** 2019-06-18

**Authors:** Xinqi Hu, Hongqi Wang, Yi Zhu, Gang Xie, Huijian Shi

**Affiliations:** 1 Beijing Key Laboratory of Urban Hydrological Cycle and Sponge City Technology, College of Water Sciences, Beijing Normal University, Beijing 100875, China; huxinqi1113@163.com (X.H.); whongqi@126.com (H.W.); 2 Shandong Academy of Environmental Planning, Jinan 250101, China; xiegang888@163.com (G.X.); shihuijian@shandong.cn (H.S.)

**Keywords:** water quality trends, spatial analysis, landscape patterns, water quality management

## Abstract

Spatial patterns of water quality trends for 45 stations in control units of the Shandong Province, China during 2009–2017 were examined by a non-parametric seasonal Mann-Kendall’s test (SMK) for dissolved oxygen (DO), biochemical oxygen demand (BOD), chemical oxygen demand (COD), permanganate index (COD_Mn_), total phosphorus (TP) and ammonia nitrogen (NH_3_-N). The DO concentration showed significant upward trends at approximately half of the stations, while other parameters showed significant downward trends at more than 40% of stations. The stations with downward trends presented significant spatial autocorrelation, and were mainly concentrated in the northwest and southwest regions. The relationship between the landscape characteristics and water quality was explored using stepwise multiple regression models, which indicated the water quality was better explained using landscape pattern metrics compared to the percentage of land use types. Decreased mean patch area and connectedness of farmland will promote the control of BOD, COD and COD_Mn_, whereas the increased landscape percentage of urban areas were not conducive to the water quality improvement, which suggested the sprawling of farmland and urban land was not beneficial to pollution control. Increasing the grassland area was conducive to the reduction of pollutants, while the effect of grassland fragmentation was reversed.

## 1. Introduction

The increasing economic growth, rapid urbanization and higher population concentrated in cities of developing countries had led to deterioration of its water bodies [1]. This water quality deterioration resulted due to point sources and non-point sources pollutants entering the water bodies, i.e., lakes, rivers and streams. Due to the dynamic changes in pollution source emissions and the spatial heterogeneity of the underlying surface, surface water quality presents dynamic and regional differences in time and space [2,3,4]. In a long period of time, the evolution of regional water quality will show a certain trend under the influence of regional pollution source structure, land use characteristics and water environmental protection measures. The trend of water quality change in a long time series can reflect the effect of regional water pollution control and water environmental protection, which is of great significance for the formulation of subsequent water environmental protection plans. The evolution trend of water quality data in long time series can be detected by using appropriate trend analysis method [5,6]. Non-parametric tests have been widely used to detect temporal trends in environmental and hydrological data, including surface water quality concentrations [7,8,9,10]. The seasonal Mann–Kendall method is an widely accepted approach to detect the temporal trend of water quality [11,12]. Previous studies on water quality evaluation and evolution trend mostly focused on river basin and urban level, but few studies were conducted at the provincial level, especially those on Shandong province in China. Shandong province is China’s largest province in terms of population and economy, ranking third in China’s GDP. The course of water environmental pollution and control in Shandong province is the epitome of water environmental protection in China. Since the reform and opening up in 1978, with the rapid development of Shandong’s economy, especially the rise of township enterprises, the water quality in Shandong has been rapidly degraded, and the average chemical oxygen demand concentration reached 260 mg/L in 1989. Subsequently, the state and the Shandong province began to pay attention to and intensify efforts to control water pollution, but the water quality was not effectively improved from 1990 to 2002. After that, Shandong continued to take water environment protection as the key point of development and adopted a series of control measures. Against this background, it is of great significance to study the changes of water quality in Shandong province in recent years. This paper adopted the seasonal Mann–Kendall method to analyze the monthly water quality data of 45 monitoring stations in Shandong province in recent 10 years, and revealed the temporal trend of river water quality and the effect of water environment protection in Shandong province in recent 10 years.

Land use plays an important role in the process of pollutant generation, migration and transformation and is an important factor affecting water quality [13,14,15,16]. Against the background of rapid urbanization in China and Shandong province, land use has been seriously disturbed. With the change of land use, various urbanization and agricultural activities, such as the increase of impermeable surface, the intensification of landscape fragmentation, sewage pipe network discharge, the use of pesticides and fertilizers, etc., have a great impact on water quality [17,18]. Identification of quantitative relationships between land use and water quality is beneficial for local managers to optimize land use and development and protect water ecosystem [19]. Modeling the correlations between land use and water quality needs to choose suitable explanatory variables [20,21]. Previous study have shown landscape pattern metrics could reflect the configuration of land use types and have been widely used as important predictors of water quality [22,23,24]. Most studies have just analyzed the percentage of land use types or the landscape pattern metrics at the landscape level with water quality, while landscape pattern metrics at the class level was paid little attention, which reflected the specific landscape configurations of each land use and could provide more targeted guidance for land use planning [25,26].

In addition, scale effect plays an important role in studying the relationship between water quality and land use. Buffer and sub-basin scale are commonly used for such analyses; however, the former have limitations in large scale application and the latter is a natural basin range, which was not convenient for practical managed [27]. The control unit is a water environment management scale with Chinese characteristics, which has been promoted and applied to local water quality protection by the ministry of ecological and environmental protection of China since 2010 [28,29]. The control units were delineated by overlaying sub-basins and administrative boundaries, with defining the administrative area responsible for each unit and national monitoring stations setting at the exit of each unit [30]. According to the water quality condition of each station, management activities such as pollution source tracing and investigation will be conducted by the corresponding administrative region to ensure that the water quality of its section reaches the standard. The downstream control unit shall only implement pollution control responsibilities within its unit, without involving the upstream unit. A total of 1784 control units were divided at the national level to help to promote the refined management. This study could fill up the blank space in understanding the relationship between landscape characteristics and water quality at the scale of control units [31].

Based on these concerns, this study applied a multiple spatiotemporal statistical approach to explore the relationship between landscape characteristics and water quality at the scale of control units in highly disturbed region. And the specific goals of this study were to: (1) identify how has the water quality changed in Shandong province in the past 10 years and its spatial variation; (2) figure out what is the relationship between river water quality and land use characteristics at the control unit scale; (3) the significant landscape factors affecting the water quality variation at the control units scale; and (4) provide effective information and references for policy makers to conduct effective water pollution control and management at the control units in China.

## 2. Material and Methods

### 2.1. Research Region

**Figure 1:**
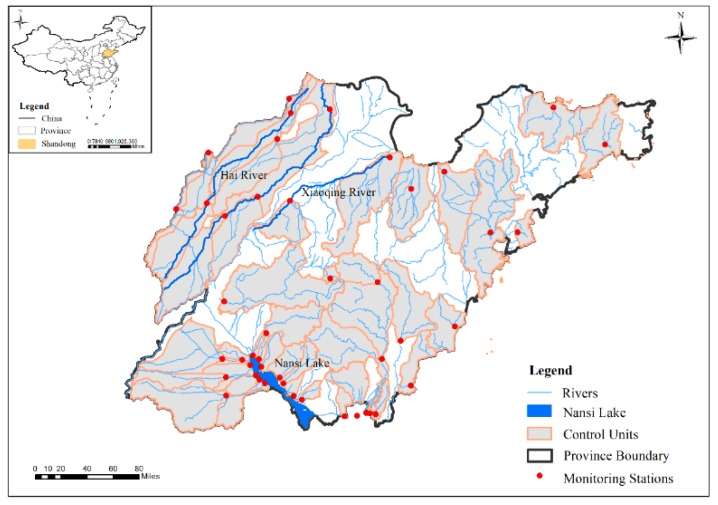
Location of water quality monitoring stations and control units.

**Figure 2:**
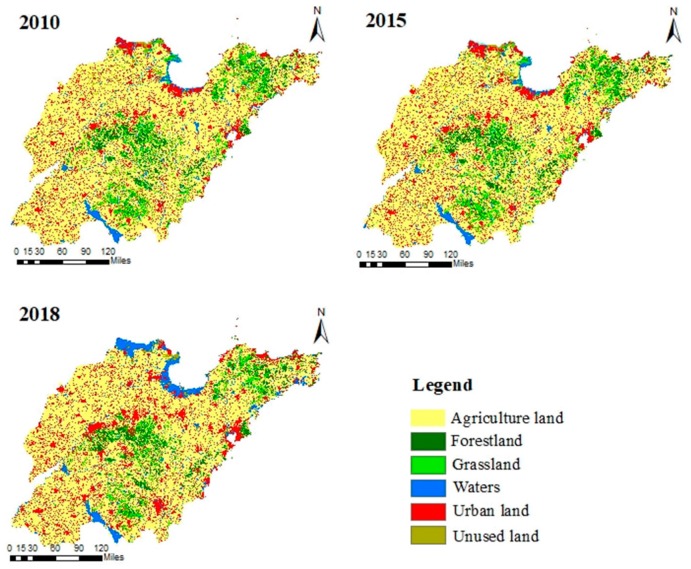
Land use classification of the Shandong province in 2010, 2015, 2018.

The Shandong province (34°22.9′ N–38°24.01′ N, 114°47.5′ E–122°42.3′ E) is a major province in China in terms of both population and economy that is located on the eastern coast and covers an area of 15.71 × 104 km^2^ (Figure 1). Abundant water systems are widely distributed in the province, with the major ones including the Hai River in the northwest, the Xiaoqing River in the middle and the Nansi Lake in the southwest [32]. The Nansi Lake, the largest freshwater lake in north China, is located within the Huai water system in the southwest portion of the province [33]. The 45 national control units in Shandong province were studied (http://www.mee.gov.cn/gkml/hbb/bgth/201602/t20160229_330936.htm), for which there were continuous water quality monitoring data during 2009–2017. As shown in Figure 1, the 45 control units were widely distributed in the province. The covered areas of control units ranged from 82.85 km^2^ to 7079.23 km^2^. Only 12 of the 45 control units likely to be affected by the upstream were concentrated in the northern and western regions, which belong to the north China plain and have relatively weak upstream and downstream influences. In addition, the Shandong province is a highly disturbed region with a dense population and an urbanization rate of 61.18%. Compared with the influence of upstream inflow, the artificial influences such as water intake, reclaimed water replenishment and sewage discharge have a greater influence on the water quality. Due to the water scarcity, the phenomenon of channel interruption often occurs. With the objective of understanding the relationship between land use and water quality on the scale of control units through statistical methods, the hydrologic and water quality simulation was not involved. Therefore, the hydrological linkage between the upstream and downstream was excluded in our research. The topography of the Shandong province is characterized by central mountainous bulges, low and flat areas in the southwest and northwest and undulating areas in the east, with elevations ranging from 2 m to 1533 m. Additionally, the province has a warm temperate zone monsoon climate, with a mean annual air temperature of 12.5 °C. The average annual rainfall is between 550 and 950 mm, with 60%–70% of the annual precipitation being concentrated from June to September. The land cover consists of 64.78% farmland, 6.24% forest, 8.39% grassland, 4.24% water systems and 15.1% urban land (Figure 2). Farmland and urban land are the principal land cover types in Shandong, indicating that the province has been highly disturbed by anthropogenic activity associated with rapid urbanization and economic development.

### 2.2. Data Description

Six water quality parameters, dissolved oxygen (DO), biochemical oxygen demand (BOD), chemical oxygen demand (COD), permanganate index (COD_Mn_), total phosphorus (TP) and ammonia nitrogen (NH_3_-N), representing physical and chemical characteristics, were selected for water quality analysis. They were the basic item for routine water quality monitoring stipulated in China’s environmental quality standards for surface water [34]. DO can reflect the pollution degree and self-purification ability of a body of water, which is essential for a healthy aquatic ecosystem. BOD, COD and COD_Mn_ were selected to represent organic matter of oxygen consumption. NH_3_N and TP were the representative variable for nutrient pollutants. The water quality data of monitoring stations at the exit of 45 control units between 2009 and 2017 were obtained from the Department of Ecology and Environment of Shandong Province. The 45 monitoring stations are the national control station and distributed in all major rivers of the province. The water quality data were reported on a monthly time scale from 2009–2017. The monthly water sample collection was conducted in strict accordance with the Regulations for Water Environmental Monitoring [35]. Laboratory analyses for the six water quality parameters were conducted in accordance with the national standard analytical methods [34]. To identify seasonal patterns of water quality data, the datasets were divided into a wet season (June–September) and a dry season (October to the following May) [21].

A raster digital elevation map (DEM) with a spatial resolution of 30 × 30 m was obtained from the Geospatial Data Cloud site (http://www. gscloud.cn) [36,37]. The DEM was used for delineation of spatial scale, and each water quality monitoring station was set to be the outlet of the delineated spatial scale [38]. The 2010, 2015 and 2018 land use data of 1 km resolutions, as well as the vector data of the water system and administrative boundary were supplied by the Resource and Environment Data Cloud Platform, Chinese Academy of Sciences (RESDC http://www.resdc.cn/) [39,40]. The land cover data were used to determine the percentage of different land cover types for each year and the percentage change of each type between 2010 and 2018. Land cover types were reclassified into six categories, farmland, forestland, grassland, water, urban land and unused land.

### 2.3. Extraction of Landscape Variables

Spatial data, including topography and land use data, were prepared for analysis. The variables describing the landscape characteristics of each control unit were extracted to identify potential factors impacting water quality parameters [41]. Metrics describing landscape composition and pattern at the class level were calculated based on land use data. Landscape composition metrics included the percentage of farmland (FA), forests (FO), grassland (GR), water (WA) and urban land (UR). A brief description of the 12 landscape pattern metrics at the class level, which have commonly been used in previous studies to explain the influence of landscape patterns on water quality, was given in Table 1 [21,42]. The land use composition of each control unit was calculated in ArcGIS 10.2 (Esri, Redlands, CA, USA) by using overlay functions based on watershed boundary and land use data layers. The landscape metrics were calculated by the FRAGSTATS 4.2 software (University of Massachusetts, Amherst, MA, USA) through land use raster data.

### 2.4. Statistical Analysis

The seasonal Mann-Kendall’s test (SMK test) is a robust, non-parametric test that can also be used when the dataset has some missing values and does not comply with any particular distribution [43,44]. To identify the temporal trends in water quality variation at each monitoring station during 2009–2017, SMK test was used with monthly water quality data to detect upward, downward, or no significant trends in DO, BOD, COD, COD_Mn_, NH_3_-N and TP [5,45].

The temporal trend values reflected by SMK’s tau had the spatial autocorrelation among stations, which could be characterized by two spatial autocorrelation indicators. Global Moran’s I was used to calculate the significance of spatial autocorrelation [46,47] and Local Moran’s I was employed to explore the spatial clusters of stations with significant high and low temporal trends, as well as stations with trends that differed significantly from surrounding stations [48]. Analyses were conducted using the weighted inverse distance and the Euclidean distance method in ArcGIS version 10.2. The Global Moran’s I and Local Moran’s Ii were calculated as follows:

(1) $$I=\frac{n}{\sum_{i=1}^{n}{\left(X_{i}-X\right)}^{2}}\frac{\sum_{i=1}^{n}\sum_{j=1}^{n}W_{ij}(X_{i}-X)(X_{j}-X)}{\sum_{i=1}^{n}\sum_{j=1}^{n}W_{ij}}$$

(2) $$I_{i}=\frac{n}{\sum_{j=1}^{n}W_{ij}}\frac{\sum_{j=1}^{n}W_{ij}(X_{i}-X)(X_{j}-X)}{\sum_{j=1}^{n}\left(X_{j}-X\right)}$$

The spatial autocorrelation indices (I) were used to compute the standard normal variate Z as follows:

(3) $$Z=\frac{I-E(I)}{\sqrt{Var(I)}}$$

where *n* is the number of stations, *X_i_* and *X_j_* refer to water quality parameters in stations *i* and *j*, respectively, *X* is the mean value of water quality parameters and *W_ij_* is a distance weight for the interaction between stations *i* and *j*. E(I)and Var(I) are the mean and variance of spatial autocorrelation indices. The Moran’s I value ranges from −1 and 1, which represents perfect negative and positive autocorrelation, respectively, while 0 indicates no autocorrelation. The local spatial patterns were classified as HH, LL, LH or HL [49]. The HH or LL patterns indicate that the particular location has significantly high or low values, respectively, while the LH and HL patterns represent the outliers surrounded by high or low values, respectively.

Pearson’s correlation analysis was conducted to explore the relationship between water quality and land use types. To quantitatively examine the linkage between water quality parameters and landscape metrics, stepwise multiple regression of water quality parameters and landscape composition and pattern metrics during dry and wet season were conducted. All regressions were performed in SPSS version (IBM, Armonk, NY, USA) at the 95% confidence interval. The significance of estimated coefficients and regression models was analyzed by *t*-tests and F-tests (F_sig_), respectively. The goodness of fit of the regression models was provided based on the adjusted coefficient of determination (R^2^). The multi-collinearity between independent variables was estimated using the maximum variance inflation factor (VIF_max_), and the model exhibited multi-collinearity at VIF_max_ > 10 [50,51].

## 3. Results

### 3.1. Temporal Trends of Water Quality Parameters

**Figure 3:**
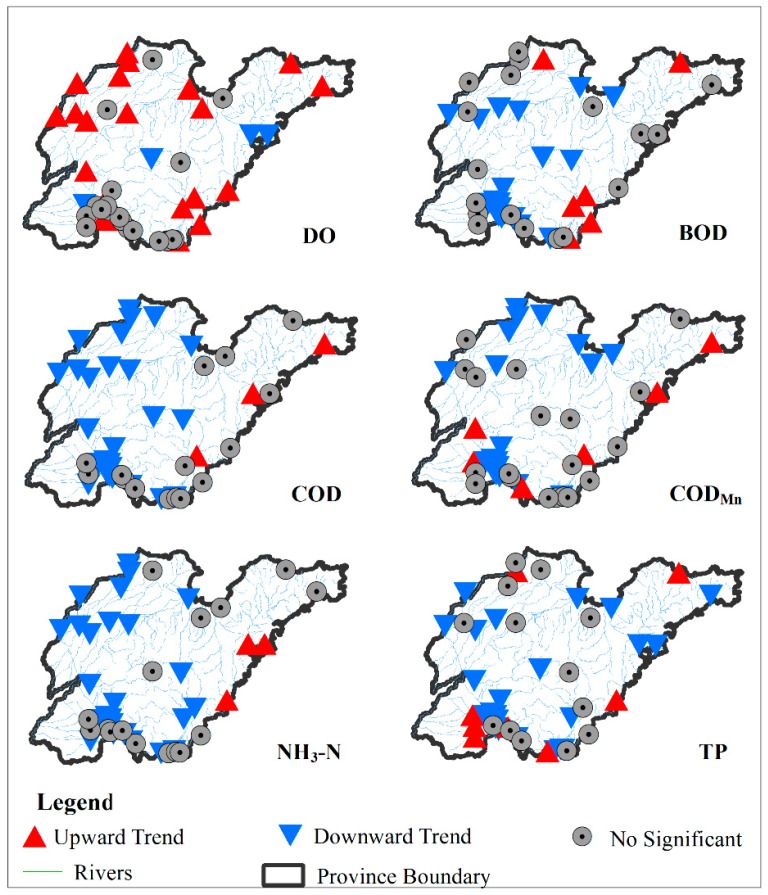
Temporal trends observed upon seasonal Mann-Kendall’s (SMK) testing for water quality parameters at 45 monitoring stations during 2009–2017.

The temporal trends of monthly water quality parameters from 2009 to 2017 were detected by the SMK test at 45 monitoring stations and revealed that the spatial patterns of water quality trends varied among these parameters (Figure 3). DO showed a significant upward trend in approximately 50% of stations during 2009–2017, while 8.9% and 42.2% of stations showed a significant downward trend and no significant trend, respectively. Except for DO, the other water quality parameters showed a significant downward trend in more than 40% of stations, while less than 18% of stations showed a significant upward trend. Especially for COD and NH_3_-N, the proportion of stations with a downward trend was close to 60%. Additionally, nearly 53% of stations showed decreasing trend for TP. Moreover, 30%–40% of the stations showed no significant trend for each water quality parameter. These findings indicated that the overall water quality situation in Shandong Province improved significantly during 2009–2017.

Based on the spatial patterns of the water quality trends, the stations showing downward trends in water quality parameters (except for TP) were mainly concentrated in the northwest and southwest of Shandong province, and especially concentrated around Nansi Lake. For TP, the stations with downward trends were not concentrated in any specific areas. For all water quality parameters except DO, the stations with an upward trend were mainly distributed in the east and south portions of the province. The stations showing increasing DO were mainly located in the northwest region. Overall, water quality improvement was greater in the northwest and southwest. In addition to TP, the water quality trends for other parameters presented a certain spatial clustering, which was further verified by spatial autocorrelation analysis.

**Figure 4:**
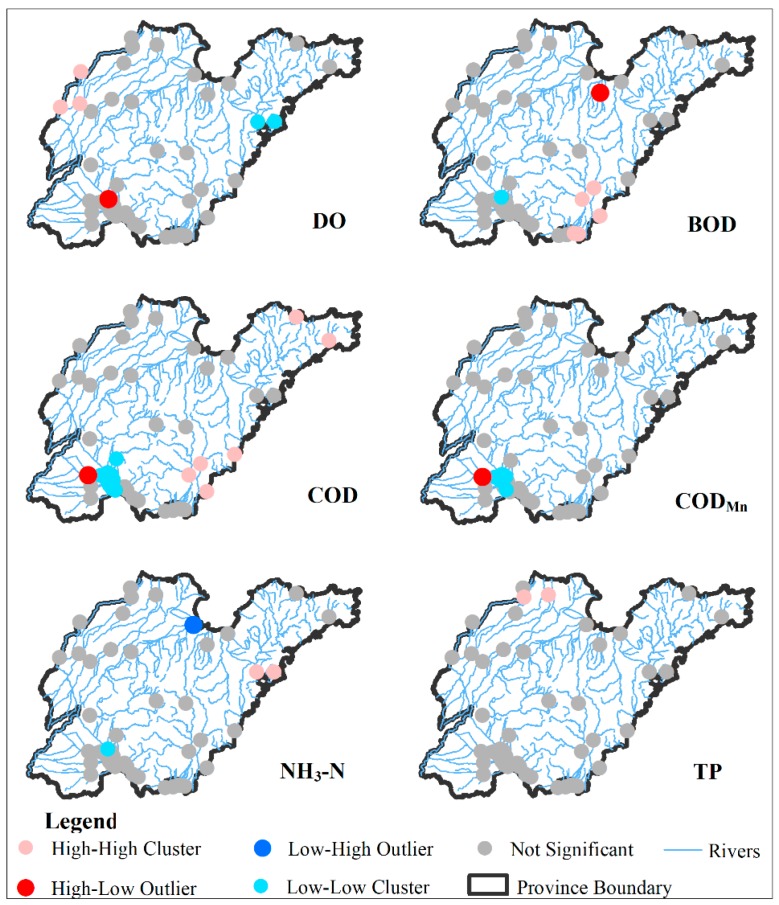
Spatial distribution of Local Moran’s I for water quality trends at 45 monitoring stations.

Most of the SMK’s tau values showed significant positive spatial autocorrelation (Table 2), with all of the Moran’s index values being greater than 0 and the Z values higher than 1.96 (except for TP), suggesting that the tau values of water quality trends were positively correlated with spatial aggregation degree. Only TP trend did not show any significant spatial autocorrelation, as indicated by the nearly even distribution of stations with significant upward and downward trends throughout the province. The Local Moran’s I analysis also revealed a clear spatial separation of trends in water quality parameters with the spatial patterns of trends in water quality parameters except for DO and TP showed consistency with the high-high clusters in the northwest and east regions and the low-low clusters around the Nunsi Lake (Figure 4).

### 3.2. Spatial Variations in Current Status of Water Quality

**Figure 5:**
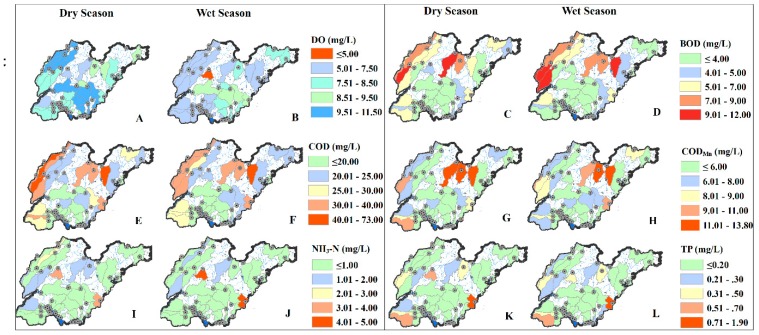
Spatial variations in water quality current status at 45 control units in wet and dry season from 2015 to 2017 (**A**: the variation of DO in Dry Season; **B**: the variation of DO in Wet Season; **C**: the variation of BOD in Dry Season; **D**: the variation of BOD in Wet Season; **E**: the variation of COD in Dry Season; **F**: the variation of COD in Wet Season; **G**: the variation of COD_Mn_ in Dry Season; **H**: the variation of COD_Mn_ in Wet Season; **I**: the variation of NH_3_-N in Dry Season; **J**: the variation of NH_3_-N in Wet Season; **K**: the variation of TP in Dry Season; **L**: the variation of TP in Wet Season).

The spatial distribution patterns of water quality current status varied with parameters and seasons [52]. Figure 5 shows the spatial and seasonal variation of the six water quality parameters in the monitoring stations of 45 control units between the wet and dry seasons during 2015–2017. The DO concentration at most stations was greater than 5 mg/L, which meets the three-level guideline of the national environmental quality standards for surface waters in China (GB3838-2002; Table 3) [53]. Water quality parameters other than DO exceeding the three-level standard indicated that the water was contaminated, while DO exceeding three-level standards indicated no pollution.

For NH_3_-N and TP, more than 60% of the control unit stations met the water quality three-level standard and the pollution levels were low. The average NH_3_-N concentration in dry season was 0.78 mg/L and higher than 0.71 mg/L in wet season. The opposite was observed for TP, which had an average concentration of 0.22 mg/L in the wet season and higher than 0.19 mg/L in the dry season. The average concentrations of BOD, COD and COD_Mn_ during 2015–2017 all exceeded the third-level standards in both wet and dry seasons, showing higher pollution levels than NH_3_-N and TP. More than 50% and 40% of the stations showed COD and BOD concentrations exceeding third-level standards respectively in the dry and wet seasons. For COD_Mn_, 37.8% and 42.2% stations had exceeded the standard in the dry and wet seasons, respectively. The average concentrations of BOD, COD and COD_Mn_ in wet season were 4.66, 23.81 and 6.55 mg/L, respectively, which were greater than 4.35, 23.72 and 6.02 mg/L in dry season. There were 20 sites with the best water quality status, and there were no water quality parameters exceeding the standard. Among these 20 sites, 12 were located at the exit of the control unit located around Nansi Lake. The analysis showed that the water quality of Nansi Lake was better than that of other areas in the province.

### 3.3. Linkage between Landscape Characteristics and Water Quality

#### 3.3.1. Characteristics of Land Use Types

The land cover types of 45 control units during 2010–2018 were extracted and analyzed to reveal the landscape composition and internal structural variations in land cover. The proportion of agricultural land and urban land in the control units was about 67% and 15%, respectively, while forestland, grassland and waters accounted for only about 6%, 8% and 3% of the total area. Agricultural and urban land were the main land cover types of the control units with the largest land percentage, indicating that the control units were highly disturbed by anthropogenic activities, such as agriculture, industry and urban living. Furthermore, the land cover percentage changed obviously during that period, with the proportion of urban land increasing and the proportion of forestland and grassland decreasing. The proportion of urban land expanded from 15.3% to 19.4% with a growth rate of 27.2%, while the reduction rates of forestland and grassland were 5.8% and 35.4%, respectively. These findings suggested that, with the acceleration of urbanization in the control units of Shandong Province, urbanized land cover increases, while ecological land decreases. For agricultural land, the proportion decreased by 1.7% and the proportion of waters increased by 16.1%. Overall, these findings suggested that the influence of landscape composition and patterns on water quality parameters at the scale of control units needs to be further investigated.

#### 3.3.2. Landscape Composition and Water Quality

To reveal the influences of landscape composition on water quality at the scale of control units, correlations between different land use types of the control units and water quality parameters of the national stations in dry and wet seasons were analyzed (Table 4). The percentage of farmland was significantly positively associated with BOD and COD in the dry and wet seasons, and positively associated with COD_Mn_ in the dry season. Forestland percentage was not significantly correlated with any other water quality parameters. Grassland was significantly negatively associated with COD_Mn_, BOD and COD in both wet and dry seasons, while it was positively associated with DO in wet season. For waters, it had a negative influence on BOD and a positive influence on DO. Urban land was significantly negatively correlated with DO and positively correlated with NH_3_-N.

Stepwise regression was conducted to identify key landscape composition metrics affecting water quality at the scale of control units (Table 5). During both dry and wet season, grasslands were found to be negatively correlated with COD_Mn_ and COD, indicating that with the increase of grassland, the concentration of these water quality parameters decreased and contamination was alleviated. The BOD was negatively correlated with both grassland and waters percentage, indicating that increases in grassland and waters will decrease the BOD concentration. DO and NH_3_-N were influenced by urban land during both dry and wet seasons, and the increasing of urban land percentage was positively correlated with NH_3_-N and negatively correlated with DO. The TP was not significantly affected by any land use types. Overall, R^2^ values calculated for all pairs of regressions were relatively small, suggesting that water quality parameters were portrayed inadequately by the landscape composition at the scale of control units and that landscape pattern metrics for each land cover type should be further investigated [51].

#### 3.3.3. Landscape Pattern and Water Quality

Landscape pattern metrics were further selected to evaluate the relationship of landscape characteristics and water quality at the scale of control units with consideration of the influence of landscape configuration and spatial heterogeneity on water quality. The results of regression results between configuration of land use patterns and water quality parameters are shown in Table 6. The overall R^2^ values were generally higher than the regression R^2^ values between land use percentages and water quality, indicating that the water quality parameters in both dry and wet seasons can be better predicted by landscape patterns than landscape composition at the scale of control units. The R^2^ values calculated for these metrics are shown in descending order in Table 7.

The largest R^2^ was observed for BOD in dry season, which was positively correlated with the mean area of water, mean Euclidean nearest-neighbor (ENN) of grassland, SHAPE of forestland and mean area of farmland, while it was negatively correlated with the Largest Patch Index (LPI) of forestland. The BOD in wet season was explained by different metrics with R^2^ 0.605. For the second largest R^2^, the COD_Mn_ in dry season was positively correlated with the mean area of waters and farmland and negatively correlated with the mean ENN and PD of forestland. For DO during wet season, the significant negative impacts originated from PLAND of urban land and SHAPE of forestland, while the positive impacts originated from CA of forestland with a R^2^ of 0.583. The other two parameters with R^2^ values bigger than 0.5 were NH_3_-N in dry season and COD in dry season, for which significant impacts originated from the mean area of urban land and forestland, COHE and ED of farmland, LSI of waters and IJI of grassland. The regression R^2^ of water quality, except for DO in dry season, was higher than in wet season. TP during dry and wet season could not be explained by the landscape composition and pattern metrics.

## 4. Discussion

### 4.1. Temporal-Spatial Variations in Water Quality

The stations showing downward trend of BOD, COD, COD_Mn_ and NH_3_-N were mainly concentrated in the northwest and southeast, especially the area surrounding the Nansi Lake, which may be attributed to the continuous and effective water pollution control actions implemented by the national and local government since 2000, including the 10th, 11th and 12th Five-Year Major Science and Technology Program for Water Pollution Control and Treatment Plan from 2001 to 2015, which were aimed at improving the water environment of several major basins in China, including the Hai River Basin and the Nansi Lake located in the northwest and southwest of the Shandong province [51,54]. The construction of new wastewater treatment plants in major urban areas and restoration projects for main water systems in the Shandong province over the past decade has resulted in significant decreases in point source pollutants discharge and improved water quality [12]. However, the water quality of control units still need improvements as indicated by the BOD, COD and COD_Mn_ concentrations at many stations exceeding the third-three level of the national surface water standard. Most of the 20 stations with no parameters exceeding the standard were distributed around the Nansi Lake, which was an important storage reservoir and water transfer channel located on the east route of the South-to-North Water Transfer Project. The good water quality of stations around the Nansi Lake was related to the many water protection activities that have been conducted in this area during the past ten years to improve and ensure the quality of transfer water [55].

The seasonal variation of water quality was mainly influenced by natural processes, such as rainfall, surface runoff and by anthropogenic activities. In this study, TP, BOD, COD and COD_Mn_ presents higher concentration in the wet season probably ascribed to the more pollutants being washed from the surface to the receiving waters by rainfall along with runoff. DO and NH_3_N in the wet season were lower than in the dry season, which may be due to increased consumption of microorganism [56].

### 4.2. Relationships between Landscape Characteristics and Water Quality

A correlation analysis between percentage of land use types and water quality showed that urban land has a negative impact on NH_3_-N and DO and farmland has a negative impact on BOD and COD, while grassland has a positive impact on these factors during both dry and wet season. These findings were consistent with the results of numerous previous studies that showed agricultural and urban land uses make negative contributions to water quality in watersheds [57,58,59], while the proportion of vegetated areas is positively correlated with water quality [60,61,62,63], which suggests the absorption and fixation effects of grassland for water pollutants [64,65]. Regression analysis revealed that increasing urban land percentage was not conductive to the reduction of NH_3_-N and DO pollution, and the increased grassland percentage was beneficial to the control of COD_Mn_, BOD and COD pollution [66,67]. The relatively small R^2^ indicated that the relationship between water quality and land use types at the scale of control units was complicated, and may need to induce the landscape metrics as well as other factors to carry out the further explanation [21].

Different from previous similar studies on sub-basin and buffer scales [68], as shown in Table S1, we found out landscape pattern metrics dominate higher relevance to water quality parameters than percentage of land use types as indicated by the generally higher R^2^ values, which was the unique conclusion we got about the relationship between landscape features and water quality at the control unit scale in Shandong province. Increasing AREA_MN of farmland will lead to increased COD_Mn_ and BOD, which may be related to the increased usage of organic fertilizer such as livestock and poultry manure and the irrigation water from production and domestic sewage [42,57]. The COHESION of farmland was positively correlated with BOD in wet season and COD and NH_3_-N in dry season. The COHESION index at the class level measures the physical connectedness of the corresponding patch type. Moreover, the ED values of farmland were negatively related to BOD in wet season and COD in both wet and dry season. Edge density (ED) represent edge length on a per unit area basis that facilitates comparisons among landscapes of varying sizes. The above results suggested that the smaller area, higher edge density and poor connectivity of farmland patches are conducive to pollutants reduction. The PLAND of urban land was negatively associated with DO in both dry and wet season, while the PLAND and AREA_MN of urban land were positively associated with NH_3_-N, indicating the adverse effects of urban land sprawl on DO and NH_3_-N [69,70,71]. Therefore, one possible approach to control the pollutants could be slow down the aggregation and sprawl of farmland and urban land and prevent it from developing in patches [72,73]. Though the landscape composition analysis suggested the positive impact of grassland percentage on water quality, the landscape pattern metrics of ENN_MN and IJI of grassland, which are the simple indicator of patch context and has been used extensively to quantify patch isolation [74], were positively correlated with BOD and COD. This finding shows that the fragmentation of grassland is not conducive to pollutant reduction.

Landscape characteristics have a greater impact in the dry season for COD, COD_Mn_ and NH_3_N, which can be seen from their higher regression R^2^ with landscape composition and landscape pattern metrics in the dry season. No landscape metrics could predict the TP concentration, which may be because in addition to landscape pattern metrics, there are other more dominant factors affecting TP, such as topography, soil properties and pollution discharges.

### 4.3. Methodology and Suggested Management

The model analysis revealed the relationship between the landscape characteristics metrics and water quality parameters, and offered a useful framework for the landscape metrics utilization to protect water environment of the control units. This study also incorporated limitations. The predictive ability of the regression models for some water quality parameters and landscape metrics was relatively low with small R^2^. This study was mainly focused on the correlation between water quality and landscape metrics, however, some factors such as streamflow, topography, soil, population density and point source pollution discharge may also affect water quality. These relativity could be taken into account in future studies to improve the prediction accuracy if the water quality prediction will be researched further [11,75].

This study demonstrated that water quality parameters was significantly influenced by landscape pattern metrics at the scale of control units and its influence on water quality was greater than landscape composition metrics. Model evaluation indicated that the control of BOD, COD and COD_Mn_ has a strong positive relationship with the mean area and connectedness of farmland and negative relationship with the edge density of farmland. In addition, the increased PLAND values of urban land had adverse effects on DO and NH_3_-N. Base on this, local management plans should take into account the configuration of land use types, such as prevent sprawling and aggregate development of farmland and urban land. The negative correlation between the grassland and water pollutants suggested that the grassland percentage may act as “sink” landscapes and should be increased appropriately, which can reduce the risk of pollution migration to water bodies [42]. Moreover, the configuration of grassland pattern can produce different effect of pollutants reduction, especially the aggregation metrics, and the fragmentation of grassland should be avoided for its positive impact on BOD and COD. Therefore, the obtained regression models can help managers to develop land use control plans that are more conducive to aquatic environments. Furthermore, the significant downward trend of water pollutants at most national stations during 2009–2017 indicated the obvious effect of pollution control measures, mainly aimed at the point sources control, taken by the nation and local government. Our study demonstrated that the point sources control should be further strengthened at the control units to improve the water quality status of national station with pollutants exceeding the third-three level of the national surface water standard. In addition to the construction of sewage treatment plants and other measures to reduce pollutant emissions, the configuration of landscape patterns conducive to water environmental protection should also be considered.

## 5. Conclusions

The SMK test for water quality parameters at 45 stations of national control units in the Shandong province from 2009–2017 suggested a significant improvement of water quality condition had occurred, with nearly 50% of stations showing significant upward trend of DO and more than 40% of stations showing a significant downward trend of the other parameters. For water quality parameters other than TP, the stations that showed downward trends were mainly concentrated in the northwest and southwest region of Shandong province, especially the area surrounding the Nansi Lake. Moreover, these stations presented significant spatial autocorrelation, demonstrating the good effects of water environmental protection in these areas in the past ten years. However, more improvements were still needed as the BOD, COD and COD_Mn_ concentrations exceeded the third-three level of the national surface water standard at more than 40% of stations. Regression and correlation analysis between land use types and water quality suggested that urban land has a negative impact on NH_3_-N and DO, while farmland has a negative impact on BOD, COD and COD_Mn_, with a significant positive impact from grassland during both dry and wet seasons, suggesting that grassland plays a role in reducing pollutants and constructing a grassland buffers of at least 20–30 m wide adjacent to the streams and lakes may be an effective measure to reduce pollutants entering rivers. Landscape pattern metrics dominated higher relevance to water quality concentration than the percentage of land use types as indicated by the generally higher regression R^2^ values. The decreased AREA_MN and COHESION of farmland and increased ED of farmland will promote the control of BOD, COD and COD_Mn_. Increased PLAND of urban land had adverse effects on DO and NH_3_-N. The increased grassland percentage produce positive impact on the pollutants reduction, while the fragmentation of grassland goes against the effect of pollutants reduction as indicated by the positive impact of ENN_MN and IJI of grassland on BOD and COD concentration. The suggestion of land use plan was concluded that the sprawling and aggregate development of farmland and urban land should be restricted and grassland act as sinks for pollutants should account for larger percentage and its fragmentation development should be avoided.

## Figures and Tables

**Table 1:** Abbreviations and descriptions of landscape configuration metrics.

Metrics Category	Landscape Metrics	Abbreviation	Description
Area and Edge metrics	Mean Patch Area	AREA_MN	The average mean surface of patches
	Total Class Area	CA	Measures the total area of all patches of the corresponding patch type
	Percentage of Landscape	PLAND	Proportion of the landscape occupied by patch type
	Largest Patch Index	LPI	Area of the largest patch of the corresponding patch type
	Edge Density	ED	Total length of all edge segments, divided by the total landscape area
Shape metrics	Area-Weighted Mean Shape Index	SHAPE_AM	A larger value of SHAPE_AM means the area is more complex and irregular in shape
	Area-Weighted Mean Fractal Dimension Index	FRAC_AM	Fractal dimension: ratio of perimeter per unit area. Increases as patches become more irregular
Aggregation metrics	Mean Euclidean nearest-neighbor distance	ENN_MN	The average distance to the nearest neighboring patch of the same type
	Interspersion and juxtaposition index	IJI	Proximity of patches in each class. High values correspond to proportionate distribution of patch type adjacencies
	Landscape Shape Index	LSI	A standardized measure of total edge or edge density that adjusts for the size of the landscape.
	Patch Cohesion Index	COHESION	Patch cohesion index at the class level measures the physical connectedness of the corresponding patch type.
	Patch Density	PD	Number of patches per 100 ha

**Table 2:** Z values of Global Moran’s I spatial autocorrelation of the trend.

Index	DO	COD_Mn_	BOD	NH_3_-N	COD	TP
Z values	4.56 **	2.23 *	3.40 **	2.28 *	4.68 **	0.73
Moran’s Index	0.511	0.24	0.37	0.24	0.53	0.06

* Significant at the 0.05 level. ** Significant at the 0.01 level.

**Table 3:** National quality standards for surface waters in China (GB3838-2002).

Parameters	Firs Level	Second Level	Third Level	Fourth Level	Fifth Level
DO	≥7.5	6	5	3	2
BOD	≤3	3	4	6	10
COD	≤15	15	20	30	40
COD_Mn_	≤2	4	6	10	15
NH_3_-N	≤0.15	0.5	1	105	2
TP	≤0.02	0.1	0.2	0.3	0.4

**Table 4:** Pearson correlation coefficients between land use types and water quality.

Parameters	Season	Farmland	Forest	Grassland	Water	Urban Land
DO	dry	0.035	0.008	0.213	0.114	−0.456 **
	wet	−0.196	0.262	0.340 *	0.317 *	−0.514 **
COD_Mn_	dry	0.304 *	−0.26	−0.407 **	−0.138	0.291
	wet	0.213	−0.185	−0.301 *	−0.15	0.249
BOD	dry	0.299 *	−0.221	−0.375 *	−0.323 *	0.233
	wet	0.373 *	−0.279	−0.408 **	−0.375 *	0.213
COD	dry	0.374 *	−0.234	−0.419 **	−0.216	0.119
	wet	0.347 *	−0.224	−0.414 **	−0.239	0.173
NH_3_-N	dry	−0.008	0.02	−0.286	−0.111	0.439 **
	wet	−0.088	0.087	−0.226	−0.04	0.426 **
TP	dry	0.037	0.011	−0.252	−0.046	0.288
	wet	0.082	−0.006	−0.245	−0.026	0.19

* Significant at the 0.05 level. ** Significant at the 0.01 level.

**Table 5:** Regression analysis of water quality parameters and land use percentage.

Parameters	Season	Function	R^2^	F_sig_	VIF_max_
DO	dry	−0.456UR	0.189	0.002	1
	wet	−0.491UR + 0.275WA	0.308	0	1.007
COD_Mn_	dry	−0.407GR	0.146	0.006	1
	wet	−0.301GR	0.070	0.044	1
BOD	dry	−0.365GR − 0.312WA	0.202	0.003	1.001
	wet	−0.397GR − 0.362WA	0.265	0.001	1.001
COD	dry	−0.419GR	0.156	0.004	1
	wet	−0.414GR	0.152	0.005	1
NH_3_-N	dry	0.439UR	0.174	0.003	1
	wet	0.426UR	0.162	0.004	1
TP	dry	—	—	—	—
	wet	—	—	—	—

WA: water; UR: urban; GR: grassland; F_sig_: the significance of F-tests; VIF_max_: the maximum variance inflation factor.

**Table 6:** Relationships between configuration of land use patterns and water quality parameters.

Parameters	Season	R^2^	CA	PLAND	PD	LPI	ED	LSI	AREA_MN	SHAPE_AM	FRAC_AM	ENN_MN	IJI	COHESION
DO	wet	0.583	FO	−UR						−FO				
	dry	0.293		−UR										
COD_Mn_	wet	0.254								WA				
	dry	0.632			−FO				WA, FA			−FO		
BOD	wet	0.605					−FA						GR	FA
	dry	0.788				−FO			WA, FA	FO		GR		
COD	wet	0.345					−FA							
	dry	0.544					−FA						GR	FA
NH_3_-N	wet	0.254		UR										
	dry	0.552						−WA	UR, FO					FA
TP	wet	—												
	dry	—												

FO: forests; FA: farmland.

**Table 7:** Regression analysis of water quality parameters and landscape pattern metrics.

Parameters	Season	Equation	R^2^	F_sig._	VIF_max_
BOD	dry	0.453 AREA_MN_WA + 0.499 ENN_MN_GR + 0.592SHAPE_AM_FO − 0.373LPI_FO + 0.256 AREA_MN_FA	0.788	0	1.568
COD_Mn_	dry	0.536AMEA_MN_WA + 0.46AMEA_MN_FA − 0.566ENN_MN_FO − 0.437PD_FO	0.632	0	2.081
BOD	wet	−0.603ED_FA + 0.483COHESION_FA + 0.341IJI_GR	0.605	0	1.309
DO	wet	−0.371PLAND_UR − 0.59SHAPE_AM_FO + 0.697CA_FO	0.583	0	1.975
NH_3_-N	dry	0.536AREA_MN_UR + 0.948COHESION_FA + 0.619AREA_MN_FO − 0.407LSI_WA	0.552	0	2.942
COD	dry	−0.533ED_FA + 0.531COHESION_FA + 0.321IJI_GR	0.544	0	1.309
COD	wet	−0.609ED_FA	0.345	0.001	1
DO	dry	−0.566PLAND_UR	0.293	0.002	1
COD_Mn_	wet	0.532SHAPE_AM_WA	0.254	0.004	1
NH_3_-N	wet	0.532PLAND_UR	0.254	0.004	1
TP	wet	—	—	—	—
TP	dry	—	—	—	—

## Supplementary Materials

The following are available online at https://www.mdpi.com/1660-4601/16/12/2149/s1, Table S1: Summary of previous studies on the relationship between land use and water quality.
