# Supplementary material for: Landscape Characteristics Affecting Spatial Patterns of Water Quality Variation in a Highly Disturbed Region
Source: Int J Environ Res Public Health. 2019 Jun 18;16(12):2149. doi: 10.3390/ijerph16122149 (PMC6617499; doi:10.3390/ijerph16122149)
Supplement: Supplementary file 1 [file ijerph-16-02149-s001.pdf]

Table S1. Summary of previous studies on the relationship between land use and water quality.

**Case Study**				**Water Quality Parameter**						**Land Use Percentage**	**Landscape Pattern**	**Other Factors**	**Author, Year**
**Research Region**	**Number of Sites**	**Spatial Scale**	**Time Scale**	**DO**	**BOD**	**COD**	**COD_{Mn}**	**NH_3-N**	**TP**				
Shandong Province (China)	45	Control Units	2009-2017	✓	✓	✓	✓	✓	✓	✓	✓	×	Our study
Zhejiang Province (China)	136	Subbasin, Buffer	2017. 09-11	✓	×	×	✓	✓	✓	✓	✓	×	Qing Gu, 2019[1]
Huzhou City (China)	34	Buffer, County	2001-2007	×	✓	×	✓	✓	✓	✓	✓	×	Rui Xiao ,2016[2]
Beiyun River Watershed (China)	25	Subbasin	2014-2015	×	×	✓	×	✓	✓	✓	×	Rainfall, Point source	Jin Liu ,2018[3]
Huai River Basin (China)	18	Subbasin; Buffer	1994-2005	✓	×	×	✓	✓	×	✓	×	Point Source	Xiaoyan Zhai,2014[4]
Hujiashan watershed (China)	15	Subbasin	2008-2012	×	×	×	×	✓	×	✓	✓	Topography, Hydrology	Y. Zhou ,2017[5]
Huai River Basin (China)	31	Subbasin	2003-2010	×	×	✓	×	✓	✓	✓	✓	×	Wangshou Zhang., 2018[6]
Huai River Basin (China)	17	Subbasin, Buffer	2000-2014	✓	×	×	✓	✓	✓	✓	×	Point source, Topography	Wei Shi, 2016[7]
Danjiangkou Reservoir (China)	9	Subbasin	2005-2009	✓	×	×	✓	✓	✓	✓	✓	Topography, Soils	L. Ai, 2015[8]
Han River (South Korea)	118	Subbasin, Buffer	1993-2002	✓	✓	✓	×	×	✓	✓	×	Topography, Soils	Heejun Chang,2008[9]
Sarapuí River Basin (Brazil)	6	Riparian Zone, Watersheds	2013-2014	✓	×	×	×	×	✓	✓	×	×	Kaline deMello,2018[10]
uMngeni Catchment (South Africa)	9	Subbasin	1987-2013	×	×	×	×	✓	✓	✓	×	×	Jean Nepomuscene Namugize,2018 [11]

References for Table S1

1. Gu, Q.; Hu, H.; Ma, L.; Sheng, L.; Yang, S.; Zhang, X.; Zhang, M.; Zheng, K.; Chen, L., Characterizing the spatial variations of the relationship between land use and surface water quality using self-organizing map approach. *Ecological Indicators* **2019,** 102, 633-643.
2. Xiao, R.; Wang, G.; Zhang, Q.; Zhang, Z., Multi-scale analysis of relationship between landscape pattern and urban river water quality in different seasons. *Sci Rep* **2016,** 6, 25250.
3. Liu, J.; Shen, Z.; Chen, L., Assessing how spatial variations of land use pattern affect water quality across a typical urbanized watershed in Beijing, China. *Landscape and Urban Planning* **2018,** 176, 51-63.
4. Zhai, X.; Xia, J.; Zhang, Y., Water quality variation in the highly disturbed Huai River Basin, China from 1994 to 2005 by multi-statistical analyses. *Science of the Total Environment* **2014,** 496, (496), 594-606.
5. Zhou, Y.; Xu, J. F.; Yin, W.; Ai, L.; Fang, N. F.; Tan, W. F.; Yan, F. L.; Shi, Z. H., Hydrological and environmental controls of the stream nitrate concentration and flux in a small agricultural watershed. *Journal of Hydrology* **2017,** 545, 355-366.
6. Zhang, W.; Chen, D.; Li, H., Spatio-temporal dynamics of water quality and their linkages with the watershed landscape in highly disturbed headwater watersheds in China. *Environmental Science and Pollution Research* **2018**.
7. Shi, W.; Xia, J.; Zhang, X., Influences of anthropogenic activities and topography on water quality in the highly regulated Huai River basin, China. *Environmental Science & Pollution Research* **2016,** 23, (21), 1-15.
8. Ai, L.; Shi, Z. H.; Yin, W.; Huang, X., Spatial and seasonal patterns in stream water contamination across mountainous watersheds: Linkage with landscape characteristics. *Journal of Hydrology* **2015,** 523, 398-408.
9. Chang, H., Spatial analysis of water quality trends in the Han River basin, South Korea. *Water Research* **2008,** 42, (13), 3285-3304.
10. Mello, K. d.; Valente, R. A.; Randhir, T. O.; dos Santos, A. C. A.; Vettorazzi, C. A., Effects of land use and land cover on water quality of low-order streams in Southeastern Brazil: Watershed versus riparian zone. *CATENA* **2018,** 167, 130-138.
11. Namugize, J. N.; Jewitt, G.; Graham, M., Effects of land use and land cover changes on water quality in the uMngeni river catchment, South Africa. *Physics and Chemistry of the Earth, Parts A/B/C* **2018,** 105, 247-264.
